# Exploring the digital extremist ecosystem: a preliminary analysis of hateful posts on Mod DB

**DOI:** 10.3389/fpsyg.2024.1502098

**Published:** 2025-02-12

**Authors:** Linda Schlegel, Lars Wiegold, Constantin Winkler, Julian Junk

**Affiliations:** Peace Research Institute Frankfurt, Frankfurt am Main, Germany

**Keywords:** gaming, modding, mod forum, extremism, radicalization, identity-based hate, Mod DB

## Abstract

The last 4 years have seen a stark increase in research on extremist activities in digital gaming spaces, particularly on gaming- and gaming-adjacent platforms. However, one area that has not received much attention so far are mod forums. While a large number of mods with hateful content have been created over the last two decades, the forums used to disseminate and discuss such mods have not yet been examined by extremism researchers. Considering the popularity of modding and mod forums among gaming communities, this is a crucial gap in our current understanding of extremist activities in digital gaming spaces. In an effort to address this research gap, this article offers an exploratory analysis of hateful and extremist posts on the popular mod forum Mod DB, including right-wing extremist, jihadist, antisemitic and mixed-ideology content. We seek to provide a preliminary glimpse into this under-researched digital space, complementing existing research on extremist activities on other gaming (−adjacent) platforms. Our research thereby broadens the current state of knowledge regarding the various gaming-related platforms frequented by extremist actors and radicalized individuals and contributes new insights about a thus far under-explored digital space.

## Introduction

Although extremist actors have sought to instrumentalize video games and gaming-related content since the 1980s, extremism studies has only recently turned its attention to the potential nexus between gaming and extremism. The issue has featured prominently on the agenda of radicalization and extremism research only since the livestreamed right-wing extremist attack in Christchurch (New Zealand) in 2019 (Macklin, 2019; Lakhani and Wiedlitzka, 2023) and subsequent attacks with a link to gaming such as the Yom Kippur attack in Halle (Germany) in 2019 (Online Hate Prevention Institute, 2019). Since then, an increasing body of research examining the various ways in which extremists of every *couleur* are seeking to exploit gaming-related content and spaces, has been amassed. Nevertheless, research into extremist activities in digital gaming spaces is still in its infancy and many open questions remain.

While the production and dissemination of propaganda games are well-known pursuits by extremist groups and have received the most media attention, recent research efforts have shown that extremist activities in digital gaming spaces are diverse and not limited to the creation of bespoke video games. This includes, among other things, producing and sharing video game modifications (‘mods’) with hateful and extremist content (Prinz, 2024) as well as the use of gaming-related digital spaces, often referred to as gaming- and gaming-adjacent platforms (Radicalisation Awareness Network, 2020; Davey, 2021). During the first phase of the RadiGaMe project (Radicalization on Gaming Platforms and Messenger Services), we conducted a literature review and brief explorations of hateful content on 20 such digital gaming(−adjacent) platforms (Winkler et al., 2024). We found that while modding is a popular activity among gamers and the prevalence of mods with hateful and extremist content is high, the forums on which such mods are hosted and discussed, are severely under-researched. The content of extremist mods has received some attention in recent years (e.g., Salvati, 2019; Prinz, 2024; Stürenburg, 2025), but examinations of hateful and extremist content in modding communities are largely absent from the current research discourse. This is a crucial gap in our understanding of the reproduction, dissemination, and reception of hateful content in digital gaming spaces, particularly because modding is such a popular activity that hateful content shared within modding communities could potentially reach tens of thousands of users.

In this article, we present an exploratory analysis of 500 posts and user profiles on *Mod DB*, one of the most popular mod forums hosting over 30,000 mods, thousands of groups, and a large forum. We purposefully cast a wide net and sought to identify relevant content across different extremist phenomena, including Islamism, right-wing extremism, antisemitism, and mixed ideology to explore the relevance of mod forums for all strands of extremism research. In doing so, this article addresses the above mentioned research gap, seeks to improve our understanding of identity-based hate in digital gaming spaces, shows that mod forums deserve more attention as crucial parts of extremist digital ecosystems and important arenas for extremism research, and aims to open further dialogue on the prevalence, nature, and characteristics of extremist content in digital gaming spaces.

## Background

Although modding has been a widespread practice since the early days of video game development in the 1980s and 1990s, “modding culture” (Postigo, 2010), the “mod scene” (Scacchi, 2010), and the communities congregating on mod platforms have not received a lot of attention from researchers so far (Thiel and Lyle, 2019; Unger, 2012). The literature on modding communities and mod forums is particularly sparse even though such forums have existed for more than two decades. There has been even less engagement with extremist activities in digital modding spaces. There are only a handful of studies focusing on mods with hateful and extremist content, although such mods have been available since the advent of the video game age. The few studies analyzing extremists modding activities are largely focused on the content of such modifications rather than the community and discussions surrounding them. Therefore, extremist, hateful, and anti-democratic communication in mod forums is a largely uncharted territory and an important gap in our understanding of modding-related extremist activities. We review both the existing state of knowledge on modding communities and extremist activities related to modding below.

### Modding

Modding refers to the creation of mods (short for modification) of existing video games. It is “the act of editing an existing video game or gaming console to change elements or produce new material and capabilities” (Curtis et al., 2022, p.220). Modding usually entails making changes to aesthetics, experiences, or structures of video games (Bostan and Kaplancali, 2010, p.1; Djaouti et al., 2010). It is one of the most popular and widespread participatory player practices in existence and is usually conducted by individuals on their own accord - mostly without the knowledge, guidance, or consent of the game developer(s) (Guajardo, 2023, p.410), sometimes with direct or indirect support from game companies (Champion, 2012, p.14f; Hawranke, 2020, p.35; see also Poretski and Arazy, 2017), and in certain cases even in the face of legal repercussions (Kretzschmar and Stanfill, 2019; Deng and Li, 2021). Modding is an example of ‘prosuming’, i.e., the shifting boundaries between media production and media consumption (Abend et al., 2020, p.2f; Dewalska-Opitek and Hofman-Kohlmeyer, 2021; Steinkuehler and Johnson, 2009).

Modding may be conceptualized as “the game equivalent of fanfiction” (Champion, 2012, p.12; see also Postigo, 2007). It stems from “the desire to tinker, to experiment with existing ideas and technologies in order to create something new” (Christiansen, 2012, p.30). Mods may satisfy players’ needs that are not met by the original game, including the desire for a customized experience that matches the players’ interests and values (Champion, 2012, p.18). Motivations to engage in modding may range from the wish for creative self-expression to the satisfaction of materialistic needs within the game (e.g., having more equipment or resources), changes made to allow for more autonomous play, more aggression, or more sexual content (Bostan and Kaplancali, 2010; Sotamaa, 2010; Tancred et al., 2020, 2024; Bilińska-Reformat et al., 2020; Deeming and Murphy, 2017).

Writing about mods of historical simulation games in particular, Salvati (2019) adds that longing for enhanced historical verisimilitude, the use of counterfactual imagination to “explore (…) fantastic scenarios,” counterfactual possibilities, and alternative realities, as well as the wish the create one’s “own, preferred vision of history” may drive users to engage in modding (p.163; see also Apperley, 2013; Whelan, 2020). Overall, modding may be conceptualized as a “counter-hegemonic force” that enables the creation and expression of content outside of established mainstream (game development) logics, perspectives, and commercial interests (Christiansen, 2012, p.39; Lauteria, 2012; Unger, 2013; but see Nieborg and van der Graaf, 2008). It is an act of player agency: “Players actively produce meaning (…) by playing with their favorite game’s algorithm to create interesting mods, and by participating in critical discussions about content and form [of these mods] in online spaces” (Salvati, 2019, p.163). Such discussions often take place in designated digital spaces, forums, and online communities.

### Modding communities and mod forums

Although mods are often created by individuals on their own accord, modding is an inherently social activity. Similar to TV series or films, whole digital communities form around games (Kocurek, 2023, p.452f) and gaming-related practices such as modding (Svelch, 2017, p.59; Abend et al., 2020, p. 3). Modding is therefore “by definition (…) a cultural activity” (Sihvonen, 2011, p. 15) and modding communities can be classified as creative communities of practice (Grace and Maher, 2021, p. 15; Thiel, 2019; Moshirnia, 2007). Mods are usually shared free of charge via digital platforms (Christiansen, 2012, p. 39) such as the game distribution platform *Steam*^1^ or designated mod forums such as *Nexus Mods*^2^, *Mod DB*^3^, *Indie DB*^4^, *CurseForge*^5^ or *Gamebanana*^6^.

The most well-researched mod-sharing site is *Steam*. However, most studies focus on the content of the mods shared on *Steam* or how they are reviewed by users rather than the user community on the platform (Moro et al., 2022; Yu et al., 2021; Lin et al., 2019; Eberhard et al., 2018; Philips et al., 2021; Windleharth et al., 2016). Only a handful of articles are concerned with social themes such as player behavior (Baumann et al., 2018), the friendship networks forming on the platform (Loria et al., 2021), and how players sanction cheating (Blackburn et al., 2011). Similarly, articles concerned with other mod forums are predominantly focused on the content, popularity, or reviews of mods rather than on the forum discussions and user interactions (Lee et al., 2020a, 2020b; Dey et al., 2016; Akbar et al., 2021; Freitas, 2021; Devanesan et al., 2023; Agarwal and Seetharaman, 2015; Guan et al., 2023, but see Owens, 2011). Despite early studies suggesting that modders have a strong sense of community (Poor, 2014), we therefore know little about these communities.

The same holds true for the mod forum we analyzed: Mod DB. Mod DB was established in 2002 and quickly became one of the most popular mod forums globally (ModDB, 2022). Despite its popularity and despite being recognized by researchers as one of the most important mod forums already over a decade ago (Christiansen, 2012; Elson and Quandt, 2016), Mod DB has rarely been the center of research efforts. Similar to other mod forums, it has featured mostly in research efforts seeking to analyze the content of mods, evaluations and rankings of mods, or has been used to recruit interviewees or survey participants to study modding activities (Pereira and Bernardes, 2022; Nielsen and Nani, 2021; Sun Lin and Chiou, 2011; Postigo, 2010; Hong, 2013). We were unable to locate research examining the user community or (topics of) discussion on the platform.

### Extremist activities in digital gaming spaces

The use of games by extremist actors predates the video game age. Already during the 1930s and 1940s, the Nazis published board games such as *Juden Raus!* (Jews Out!) in an effort to provide ‘playable’ propaganda (Morris-Friedman and Schädler, 2003). Since the advent of the digital age, extremists are increasingly seeking to instrumentalize online gaming, gaming-related content, and gaming-related platforms for their ends (for an overview see Schlegel and Kowert, 2024; Radicalisation Awareness Network, 2020). Both Islamist (Mahmoud, 2022; Lakomy, 2019; Winkler, 2024) and right-wing extremist actors (Wells et al., 2024; Prinz, 2024) do so in various ways, including by producing bespoke propaganda games (Robinson and Whittaker, 2021), exploiting existing games by developing modifications or seeking to make use of in-game communication features (Radicalisation Awareness Network, 2021; Kowert et al., 2022), using gaming and gaming-adjacent digital platforms such as *Steam*, *Discord*^7^, *Twitch*^8^, and *Roblox*^9^ (Schlegel, 2021c; Anti-Defamation League, 2020; Davey, 2021, 2024; Moonshot, 2024; Winkler et al., 2024), appropriating video game references and aesthetics (Kingdon, 2023, 2024; Dauber et al., 2019), as well as implementing gamification elements (Schlegel, 2020, 2021a, 2021b; Lakhani, 2024).

While the production of bespoke games by various extremist organizations, including right-wing extremist and white supremacists such as the National Alliance as well as Islamist organizations such as Hibollah and the so-called Islamic State (ISIS), has received considerable (media) attention, the creation of mods with hateful and extremist content is a much more widespread yet lesser known activity than the development of propaganda games. So much so that Salvati (2019) postulates extremist modding “has become something of a cottage industry in recent years” (p.156). Although mods with explicitly Islamist content exist - including, for instance, by Al-Qaeda supporters who infamously modded the video game *Quest for Saddam* into *Quest for Bush* in the early 2000s (Schlegel, 2020, p.7) - the overwhelming majority of research efforts on extremist modding activities focuses on right-wing extremist mods.

Already in 2010, Selepak showed that most games distributed and advertised on right-wing extremist and white supremacist websites were mods of popular video games at the time, in which the original enemies were replaced with ethnic or religious minorities (see also Daniels and Lalone, 2012). There is also a ‘tradition’ among right-wing users to modify strategy games, particularly those set during WWII, to allow players to assume the role of Nazis and win the war with the Wehrmacht (Davey, 2024, p.103; Prinz, 2024, p.67). Today, extremist modifications are distributed either via designated digital platforms such as mod forums or via private communication channels such as *Telegram* (Global Project Against Hate and Extremism, 2024). However, their content does not seem to have changed considerably: These mods still largely target religious or ethnic minorities or allow players to act out Nazi and white supremacist fantasies (Khosravi, 2017; Wells et al., 2024; Maisenhölder, 2018).

Stürenburg (2025) asserts that there are three main types of right-wing extremist mods: total conversions, mods adding right-wing extremist content and symbols, and mods deleting ‘unwanted’ game elements. To create total conversions, modders employ the basic mechanic and engine of a given game but otherwise create an entirely new game. Well-known examples are various right-wing extremist mods of *Doom II*, in which players can assume the role of Hitler or other right-wing figures and shoot Jews or People of Color (PoC). The insertion of right-wing extremist content is the most popular type of right-wing extremist mods and includes, for instance, mods allowing players to wear SS-uniforms, build concentration camps or play Wehrmacht music. Lastly, some users delete ‘unwanted’ content such as PoC or trans characters or symbols such as pride flags so that they can play undisturbed by ‘woke’ content and the game matches their personal beliefs. Such hateful mods are apparently particularly popular with players, who wish to act out fantasies of superiority and control (Salvati, 2019).

However, current research does not contain a lot of information on the reception and discussion of such mods. Only anecdotal evidence on user comments and hateful posts in digital mod spaces is available. Several researchers report a selection of user comments indicating the favorable reception of mods with extremist content. Ebner (2019), for instance, quotes a user expressing their excitement about the right-wing extremist mods of *Millennium Dawn*, because of the focus on “racist supremacy” within the gameplay and because there are “so many options for genocide, a lot of fun” (p.143, our translation). Similarly, Salvati (2019) describes the favorable reception of a *Europa Universalis IV* mod titled “Fall of Islam.” It allows the simulation of wars of religious extermination against Muslims and some users commented approvingly: “Damn, why is not the world like this?,” “No muslims = better world” or “Removes kebab, I love it” (p.155f). The neo-Nazi website Stormfront also hosts several discussions on right-wing extremist mods and even offers a ‘wishlist’ that allows users to suggest new mods (Khosravi, 2017).

Such comments are anecdotal evidence of the appeal of extremist modding activities and the importance of the discussions surrounding them. However, communities on mod platforms where such extremist mods are being shared have not featured prominently in studies on gaming and extremism so far–with the exception of some right-wing extremist (modding-related) communities on *Steam* (Newhouse and Kowert, 2024; Davey, 2024). To be sure, there is growing evidence that extremists of every ideological background are active on various gaming and gaming-adjacent platforms (Winkler et al., 2024; Davey, 2021; Schlegel, 2021c). Surveys of gamers and users of gaming (−adjacent) platforms revealed that hateful content, including extremist tropes and narratives such as holocaust denial, white supremacy, racism, and LGBTQAI+ hatred are so widespread in gaming communities, that even users without any connection to hateful actors are very likely to witness such identity-based hatred (Kowert et al., 2024; Schlegel and Amarasingam, 2022; Olaizola Rosenblat and Barrett, 2023; Anti-Defamation League, 2021, 2022, 2024). Not all of this content is posted by extremist actors or radicalized individuals, but these actors may benefit from and strategically exploit the prevalence of hate and toxicity in digital gaming spaces to spread their worldview and normalize extremist narratives (Wallner et al., 2023; Munn, 2023). However, despite growing research on extremist activities in digital gaming spaces and extremist mods, mod forums and the communities on these platforms have received little attention from extremism researchers.

Reisinho et al. (2024) argue that “unethical mods,” including those with hateful and extremist content, are a major challenge and one of the most important gaps in our current understanding of modding activities (p.878). We argue that not only these mods as such but, particularly, the modding communities hosting and debating these mods as well as content shared in the discussion threads of mod forums are also a crucial research gap that deserves attention.

## Methodology and data collection

### Case study selection

In this paper, we focus on the analysis of hateful content on the modding platform Mod DB. In the first phase of the RadiGaMe project, we explored 20 gaming (−adjacent) platforms to gain insight into the platforms’ functions and characteristics, potential restrictions for research efforts (e.g., no search bar or mostly private communication channels), known links to extremist activities, and prevalence of openly accessible hateful and extremist content. This exploration revealed that mod platforms have not featured prominently in research on extremist activities despite the fact that hateful and extremist content can be located in these digital spaces with relative ease. We determined that not only the modifications themselves but particularly the discussion areas of the mod forums featured relevant content and could be researched via openly accessible search features (Winkler et al., 2024; Winkler and Wiegold, 2024). Mod DB stood out as particularly relevant, promising, and relatively easy to research during this exploration.

In addition, Mod DB is a very popular platform, indicating that high numbers of users could potentially be exposed to hateful discourses on the platform. Although user statistics are not available for the platform, the sheer quantity of mods and interactions suggests that it is a highly frequented platform. At the time of writing in July 2024, the platform hosts over 31,000 mods. The platform is also currently home to almost 37,000 groups, some of which contain thousands of posts, and a forum area with an additional 580,000 posts. Users can also comment on mods and write reviews, both of which are not included in the numbers above. Depending on their popularity, mods can have several thousand comments and reviews. In other words: The potential corpus of data on user interactions and posts on Mod DB is enormous.

Overall, we selected Mod DB for this study, because of its apparent popularity, the platform characteristics that make it relatively easy to search its content, and our preliminary exploration, which suggested that Mod DB hosts considerable amounts of hateful and extremist content. We therefore believe that the platform may be highly relevant for extremism research and an analysis of Mod DB lends itself to open a discussion about extremist activities in digital modding spaces.

### Data collection and analysis

Between June and August 2024, we collected 500 of publicly accessible posts and profiles on Mod DB via keyword searches. We sought to cover several types of ideological content, including Antisemitism, racism, conspiracy narratives, right-wing extremism, white supremacism, Islamism, and jihadism. Search terms included words like Hitler, SS, MAGA, Muslim, Taliban, ISIS, Israel, Zionist, ZOG and similar terms known to be used in extremist circles. The oldest post dates from November 19 2008, the most recent post in our sample was posted on July 28 2024. Four hundred and seventy seven posts in our sample are English language posts, 14 are in Arabic, 5 in German, 1 in Yiddish, 1 in Spanish, 1 in Turkish and 1 in Polish.

Although all posts and profiles we examined are publicly available and Mod DB can be regarded as a public digital space, we nevertheless seek to preserve the anonymity of the users as much as possible. We follow Boyd (2015) call to “use digital content to convey impressions without directing attention to specific people” (p. 91) and report quotes without attributing user names unless the user name itself conveys hateful messages, e.g., by reproducing right-wing extremist codes.

We conducted a qualitative content analysis (Mayring, 2022) using MAXQDA. The codebook was developed using a mixed deductive-inductive approach. We first developed a list of rudimentary deductive codes. These included user information, general content categories, stylistic aspects, the degree of radicalization (as far as it was possible to judge for the coders) as well as political and ideological tendencies and concepts. Then, we added inductive codes as they appeared during the coding process. The process of generating inductive codes was particularly important for this study due to the lack of prior knowledge and research into the nature and patterns of hateful posts and extremist content in mod forums. Amendments to the codebook were shared immediately with all coders, ensuring the swift adaptation of all codes to the data set and minimizing the need for additional rounds of re-coding. To ensure inter-coder reliability, we coded the first 50 posts together as a team before distributing the data among the coders. At later stages, if coders encountered posts they were unsure about, we also coded these posts as a team. The codes are not mutually exclusive and several times posts contained references to multiple ideologies. The full codebook can be found in the Supplementary material.

### Content for the ‘lulz’

It is sometimes argued that hateful digital content, particularly content posted in digital gaming spaces, is particularly difficult to analyze, because users may post content merely ‘for the lulz’, i.e., to mock and provoke other users. In these cases, the posts are not necessarily indicative of any ideological beliefs but rather a form of ‘joking’ and ‘shitposting’ (Salvati, 2019, p.159f). We included a code on “suspected trolling,” but recognize that this is a subjective impression and mainly speculation. Often, we were unable to classify posts unambiguously as we do not know the user’s intention. We therefore make no argument about the potential radicalization of individual users.

However, we nevertheless classify these posts as hateful and inherently problematic for two reasons: Firstly, an antisemitic post is a display of Antisemitism - no matter whether the user’s intention is to make a joke or express their personal beliefs. Therefore, we can reasonably categorize such posts as hateful. Secondly, extremist actors may deliberately make use of ‘dark humor’ and ‘shitposting’ to insert their narratives into mainstream discourses and normalize their ideas (Fielitz and Ahmed, 2021; Nagle, 2017). Salvati (2019) reports that the founder of the neo-Nazi website *Daily Stormer* specifically mentioned this type of posting behavior as a strategic course of action for the alt-right and suggested “non-ironic Nazism masquerading as ironic Nazism” as a viable tactic to increase the proliferation of right-wing extremist content and influence digital discourses (p.160). Some authors even postulate the “collapse of the extremist/troll boundary,” in which ‘jokingly’ verbal abuse becomes a key element of digital extremism (Munn, 2019). This means two things: We cannot exclude the possibility that even though a post may appear to be ‘shitposting’ it may nevertheless be used to deliberately promote hateful content. In addition, even if this content is posted merely ‘for the lulz’, it nevertheless contributes to the normalization of hateful ideas on Mod DB. Therefore, while we do not argue that any given post is indicative of the user’s radicalization, these posts deserve attention in their own right as they are reproducing, promoting, and potentially normalizing hateful narratives.

### Limitations

There are several limitations of this study, mostly pertaining to access and, therefore, data selection. We faced the following access issues:

- Certain information, such as a complete list of user profiles, is only available after having been an active member of Mod DB for a long period of time. We created new accounts to carry out this study and did not actively participate in discussions. Therefore, we could not access such information and the data consists solely of publicly available posts and profiles users can access immediately after sign-up.
- The data on Mod DB can be deleted and edited. It can therefore not be ruled out that highly relevant posts have been deleted or modified, either by the users themselves or by moderation efforts, which potentially skewed our data set.
- While we found no evidence to suggest that profiles included in our sample were fake or controlled by bots, we cannot rule out this possibility entirely. However, we believe that if such profiles are present in our sample, they are a small minority as most posts were deemed genuine by the coders.
- The data protection and collection guidelines of the RadiGaMe project forbid us to access closed, non-public areas of the platform. Therefore, we make no statement about the nature of the content in such areas of the platform and report solely openly accessible data.

In addition, we have specifically searched for the posts and user profiles in our data set and, hence, cannot assess how likely it is that the average user on Mod DB stumbles upon such hateful content or estimate how many users are exposed to such content regularly. Similarly, we acknowledge that we analyzed a mere 500 out of hundreds of thousands of posts on Mod DB. Therefore, our findings cannot be generalized to all communication on Mod DB and we make no claim about the relevance of these posts in relation to the vast majority of (non-hateful) posts on the platform. We do not believe that these limitations distort or skew the findings in a decisive manner or pose an issue to the goal of our exploratory study, namely to provide a first glimpse into identity-based hate and extremist content on Mod DB.

## Results

In the following section, we describe the results of an analysis of 500 posts on Mod DB, sorted by phenomenon. We present findings related to Islamist, right-wing extremist, and antisemitic content on Mod DB as well as posts displaying mixed ideologies and ambiguous narratives.

### Islamism

We coded 70 posts as connected to Islamism, 21 as Salafist, and 38 as jihadist. Most of the posts were shared by a limited number of users, suggesting that Islamist content was either moderated more effectively than other types of hateful content or that not many users shared such content on Mod DB. The author coding the Arabic posts in our sample observed that several profiles posted in both English and Arabic, using English statements for benign commentary but switching to Arabic when posting slurs or expressing explicit hate and extremist sentiments. This could indicate that some users seek to avoid moderation by switching to non-English posts whenever they deem the statements as potentially drawing attention from moderators.

We were able to locate user profiles supporting jihadist activities and a violent interpretation of the Qur’an, references to Islamic songs (nasheeds) such as *Saleel al-Sawareem* (Clashing of the swords) which was used in propaganda and beheading videos of the so-called Islamic State (ISIS), comments on the Gaza war and anti-Israel sentiments, glorification of organizations such as Hezbollah, and a post potentially related to a failed ISIS attack. For instance, in a group dedicated to Islamic topics, we found the following post:

*“You can say what you want. But Islam is Islam, and it’s an obligatory duty upon Muslims to spread it by word and sword, and I’m retaining my rights to practice my religion in this way. Islam is religion of war and not peace, and if you do not believe me, read the Qur’an.* [*sic*!]*”*

Similarly, under a *Battlefield II* modification allowing players to assume the role of the Taliban, a user commented:

*“The jihad is good. We muslims aren’t doing anything wrong. We did not attack US or Israel first, they did attack us first. There is nothing wrong with defending our countries and faith through Jihad! And if u have brains you have to know that. The US came to our countries to kill and destroy and murder innocent people. The Jihad and Mujahideen are good, they are the real freedomfighters who fight for Allah against the Kafireen. So shut up and support the Jihad, and Mujahideen Btw, if Mujahideen kill innocent people, if, its with a reason, not like the US, who just kills people cause they like it*… *We have to defend our Islam against the agressors who came to our countries for oil and money…*[*sic*!]*”*

We also found explicit references to anti-Israel sentiments, antisemitism, and the war in Gaza, including users reporting that they *“have fun”* killing Jews in modifications, threats that they *“will retake both the dome and the aqsa soon”* which refers to the al-Aqsa mosque and dome of the rock in Israel, and statements such as.

*“Hello, just wanna say the Jihad will destroy Israel and restore Palestina in the near future*… *just that you know it…*[*sic*!].*”*

Our dataset also included references to and reproduction of ISIS propaganda material. For instance, users discussed mods of first-person shooter games allowing players to assume the role of ISIS fighters, and commented on these mods with explicit depictions of violence in both photo and video format, including executions, mass rapes and dead children allegedly killed in the war in Syria. One of the profiles that stood out was an account frequently sharing ISIS-related extremist posts and claiming to have joined ISIS and traveled to Syria in 2014. The profile was used to keep a “diary” and document the user’s experiences and impressions in Syria. However, we were unable to verify whether this user had actually traveled to Syria and the diary was authentic.

We also found a post potentially related to a failed ISIS attack in Garland, Texas in 2015. In a group used to discuss topics surrounding Islam, the Qur’an, and exchange greetings for holidays, we located the following post:

*“The infidels are hosting a contest to draw blasphemous pictures of our beloved prophet (honor and peace be upon him) in garland, Texas (…) Muslims in the area should go and teach them a lesson like the lesson given to Charlie Hebdos crew. And do not try to say this is unislamic. The Messenger of Allah himself called out Muslims to assassinate a famous poet of that time who was making insulting poetry against him. So there’s no excuse* [*sic*!]*”*

This was posted on 2nd of May 2015, less than 24 h before the Curtis Culwell Center attack, a failed ISIS-inspired attack on an art contest in Garland, Texas that saw two perpetrators shoot an unarmed security guard before they were shot by police. The post was met with a lot of support, including a high rating and many approving comments stating, for instance *“enough is enough”* and that it is *“time to hit back.”* We could not determine whether the users involved had any knowledge of the attack plan, but the post shows that even local events were met with calls for ISIS-inspired violence in certain groups on Mod DB.

### Right wing extremism

Most research on gaming and extremism focuses on right-wing extremist content in digital gaming spaces. We also found a substantial number of right-wing posts on Mod DB. Hundred and fifty seven posts were coded as right-wing extremism, 47 as borderline content. Of these, 63 posts included a glorification of National Socialism (NS), 49 espoused nationalism, and 28 advocated for ethno-pluralism. A large number of relevant profiles, including user profiles with relevant profile pictures such as black suns and names that could unmistakably classified as right-wing extremists such as 卐BlitzKrieg卐, could also be located. Unsurprisingly considering the research discussed above, we found several mods with right-wing extremist content such as a mod titled *Totaler Krieg 1939–1945* (Total War 1939–1949) and several Call of Duty mods re-imagining the events of WWII, including on the liberation of Auschwitz and the experiences of German soldiers.

The most important themes that emerged from the content analysis were NS glorification and racism. In a group called *Wehrmacht Gamers*, right-wing extremists images, videos and comments were found. The group is tagged as ‘educational’ and the founders frequently pointed out the educational character of the group. Nevertheless, we located a substantial amount of Nazi propaganda, ‘*Sieg Heil’* statements, and references to the Nazi propaganda song *Horst Wessels Lied*. Several users expressed clear disagreement when they were reprimanded for not following the alleged educational character of the group.

*“(…) Bla Bla Bla another nazi on MODDB Bla Bla Bla Bla (…) You can accuse me of what you want for tell the truth.* [*sic*!]*”*

Similarly, in the group *Conservatives of Mod DB*, National Socialist policies were glorified, particularly in relation to the standing of women in society and reproduction incentives:

*“I am 99% sure that the NatSoc’s heavily supported the family and provided incentives for large families. (*…*) Women where encouraged to become mothers. NSDAP offered rewards for women who had more than 1 children. Women who had 5 or more children where given awards by the state.* [*sic*!]*”*

The same user also comments favorably on Hitler in particular and, indirectly, on his fight against communism and the Jewish elite:

*“There really is no possible way to stop the awakening occurring. That Hitler was fighting against the worst evil the world has ever seen, the international (((finance))) system and (((communism))). The NatSoc’s also outlined how the achieve a better society.* [*sic*!]*”*

The triple brackets, so-called (((echos))), are used in antisemitic and conspiracy circles to identify alleged or actual Jewish actors to suggest these actors are driven by (hidden) ‘Jewish interests.’ In addition to NS glorification, racism emerged as a central theme. This included both overt racism against PoCs and an alleged racism against white people, a common white supremacist narrative. For instance, a *Europa Universalis IV* mod, in which players can re-enact the Rhodesian Bush War (the Zimbabwean war of independence 1964–1979), received overwhelmingly positive feedback, including racist statements such as:

*“The Mod is great, i can finally release the devil within me and start killing niggas. Thanks you made my day fam* [*sic*!]”

The mod creator replied in kind, alluding to an alleged anti-white propaganda that fueled the war:

*“Haha sure, hope you enjoy my little mod! It should be noted however that the Bush War wasn’t fought because of racial tensions but because of Communist insurgents armed by the Soviet Union, who later on in the war started spreading anti-white propaganda across Rhodesia to make the blacks hate the whites to help bring down the government.*:*).”*

The comment illustrates the historical revisionism postulated by right-wing extremist narratives and possibly the wish of the mod creator to enact counterfactual historical realities in accordance with their own view of the event, a key driver of right wing extremist modding activities (Salvati, 2019). Similarly, when a user pointed out the display of race relations in a *Fallout 4* modification titled *Grounded Commonwealth*, which was advertised as explicitly ‘non-woke’, the comment was met with resistance and suggestions that the user simply did not appreciate the ‘real’ and accurate historical setting:

*“I’m sorry that the 1950s is too racist for you (*…*) too bad nobody else cares* [*sic*!]*”*

Supposed anti-White propaganda efforts and even racism against white people also emerged as an important issue in the data overall. We encountered several users complaining about the ‘excessive’ diversity in video games and the erasure of white characters in video games. For instance, a user named Erwin Rommel (a German general in WWI and WWII), complained:

“*All the people you kill are white men too, but I bet if that’s all the mod did then you would not be in here whining about it, in fact you’d probably give it a 10/10 and advertise it to your cuckfriends* [*sic*!]”

In other words: Players in the mod kill both white and PoC Non-Playable Characters (NPC), but if the mod only allowed for the killing of white characters, it would be reviewed approvingly, particularly by the LGBTQ community. This suggests that the user believes killing white NPCs is received favorably by other users, whereas the killing of PoC characters is condemned. Similar fears apparently drove the development of a *Stellaris* mod titled *European Phenotypes*. The creator describes their reasons for developing the mod as follows:

“just a mod that turns human pops European. Zogs, snowflakes and xenos can kiss my a***s. Since there are no more white or Europeans in this game, this mod brings them back. (…*) I am just sick of seeing whites erased everywhere.* [*sic*!]*”*

Because the original game allegedly no longer includes White or European characters, the modder turned the whole human population in the game into white Europeans despite anticipated resistance from “zogs” (Zionist occupied government, a prominent right-wing extremist conspiracy theory postulating Jewish world domination) “snowflakes” (progressive and allegedly easily offended individuals) and “xenos”(non-White, non-European people). Other users commented favorably, for example: “F**k White erasure. Thanks for making this.”

### Antisemitism

In the course of data collection, antisemitism emerged as a central, cross-cutting phenomenon. 71% of the posts were classified as antisemitic. Antisemitic posts were found across different ideologies, including user profiles displaying right-wing extremist attitudes as well as profiles espousing Islamism. Although antisemitism often emerges in subtle ways, we coded mainly overt and unmistakable expressions of antisemitism. These included stereotyping, holocaust denial, Israel-related antisemitism, and posts related to Hamas’ attack on Israel on October 7, 2023.

We found several mods openly advertising classical antisemitic stereotypes. For example, the preview image of a mod for the first person shooter *S.T.A.L.K.E.R.: Call of Pripyat* (GSC Game World 2009) shows a modified NPC with a Star of David necklace and payos (side curls). The character is advertised as speaking “*in a light Jewish accent*.” There are several favorable comments on the mod, often affirming antisemitic stereotypes:

*“Make his nose bigger in the next update.”*

*“does he steal your money?”*

Another form of antisemitism that repeatedly comes to light on the platform is post-Shoah antisemitism (i.e., holocaust denial), e.g.:

*“holocaust was a big hoax* [*sic*!]*”*

The term “*hoax*” implies that not only did the holocaust not happen but that it was invented for political gain. Another user added:

*“The Holocaust has nothing to do with the Jews only […] Whether Zionist propaganda has appropriated the issue and the Term.”*

Jews and the state of Israel are implicitly accused of benefiting from the Shoah. Under a picture showing Adolf Hitler, one user comments:

*“he lied with his holocaust (which never happened) to give the zionist leaders of the world the reason to create israhell* [*sic*!]*”*

Unsurprisingly, Israel plays a central role in antisemitic content on Mod DB. Almost 67% of antisemitic posts and 48% of the posts overall were Israel-related. Israel-related antisemitism is by far the most widespread form of antisemitism today (Schwarz-Friesel, 2019, p. 311), which is also reflected on Mod DB. Israel-related antisemitism manifests itself in various forms on the platform. Many posts that exhibit Israel-related antisemitism also contain references to classic antisemitic, dehumanizing narratives, for example:

*“Israelis are parasites.”*

Similarly, we found several user profiles with antisemitic descriptions such as:

*“For Zionests: We will keep struggling and fighting until ALL historical Palestine is ours AGAIN!!!* [*sic*!]*”*

This post delegitimizes Israel’s right to exist and calls for its complete destruction. A phenomenon that is also expressed in the widespread slogan “*From the River to the Sea, Palestine will be free*,” which is also repeatedly taken up on Mod DB, as well as comments such as.

*“FREE GAZA FREE PALESTINE AND FREE THE WORLD FROM ZIONISM*!! [*sic*!]*.”*

Israel-related antisemitism is expressed, among other ways, through the demonization of Israel, for example:

*“You cant denie Israel took Muslim lands, fight with Muslim people, treat the Palestinians now, like the Jews/Israelis were treaten by the Nazis. Israel is the agressor and the oppressor.*

*Long live the lions of Hamas!!!* [*sic*!]*”*

Hateful posts were sometimes aimed directly at individual users:

*“you fuckin zionist scum* [*sic*!]*”*

*“Garbage Trash Cringe Ass Bad Shit Israeli Mod.* [*sic*!]*”*

*“hamas did not frag ur stupid ass so you stop spamming this crap here? no such luck i guess* [*sic*!]*”*

The post contains the term “*frag*,” which is common in the context of first person shooters and is transferred here to the real-world murder of a user. Modifications that allows users to play Israeli actors such as the Israel Defense Forces (IDF) were attacked particularly frequently. There are several posts of this type under one such mod:

*“OY VEY SHUT IT DOWN!!!!* [*sic*!]*”*

The poster ironically uses the Yiddish “*Oy Vey*” to express their dislike. The rejection of Israel is even more evident in other contributions to the mod in question:

*“Fight for occupation instead of independence.”*

Some users resort to particularly drastic accusations in their comments:

*“Perhaps we’ll kill civilians and displace people from there homes, that’d be great fun for the IDF. Wonder if you can drive a bulldozer and destroy some poor family’s house just before the winter hits. Maybe after the fight for independence is done we can see an expansion pack* [for the mod] *called ‘invade all your neighbors’* [*sic*!]*”*

Israel-related antisemitism in particular also featured references to current events. The developer of the antisemitic game *Fursan al-Aqsa: The Knights of the Al-Aqsa Mosque* not only released an expansion following Hamas’ attack on October 7, 2023, which made it possible to assume the role of a Hamas member, but also other updates, such as a so-called “diapers” update, which gave IDF soldiers diapers with red triangles, used by of Hamas. This game, as well as the updates, seemed to be very popular on Mod DB.

We also found users mixing antisemitic sentiments with other issues. For example, one user published several images in which various left-wing groups are insulted in an antisemitic manner.

These included one in which icons of communism were marked with Stars of David. Christian interpretations are also used. The Magen David is described as follows: “6 Points, 6 Triangles, 6 Lines = 666”. Jews are interpreted here as the Antichrist, which is a traditional antijudaic phenomenon. The devil himself is described as a Jew in the picture: “*THE DEVIL IS A JEW AND HIS CHILDREN ARE COMMUNIST JEWS*”. The picture closes with a call for murder: “‘Christian’ Zionists should be shot as Traitors!”.

In this image, the religious demonization of Jews blurs with the antisemitic and anticommunist narrative of the idea of Jewish Bolshevism, which accuses Jews of leading a communist conspiracy to harm either the West or right-wing nationalists. The Nazis already used this myth to justify the Shoah.

Other users affirmed such sentiments, e.g.:

*“Hezbollah will hunt you bolshevik scum! Long live Golden Dawn!”*

The user explicitly approves the actions of two vastly different antisemitic organizations with opposing ideological backgrounds, the Lebanese Islamist terrorist organization Hezbollah and the far-right Greek party Golden Dawn. The user himself is aware of this seemingly contradictory constellation:

*“I did not say Hezbollah and Golden Dawn are the samething but, I support both of them because they are both anti-Zionist* [*sic*!]*”*

The positive reference to the quite different organizations is justified by antisemitism that unites them.

Even if the motivation behind the statements is not always clear, a contextualization of the posts and profiles of the communicating users often suggests right-wing extremist or Islamist sentiments. However, there are also some antisemitic posts that can be relatively clearly attributed to the radical left. In 2016, for example, one user posted a propaganda video featuring armed members of the antisemitic Marxist-Leninist Popular Front for the Liberation of Palestine (PFLP) and its spin-off, the Marxist-Leninist and Maoist Democratic Front for the Liberation of Palestine (DFLP). Another apparently left-wing user, who repeatedly ascribes himself to an anarchist movement draws a Nazi analogy to Israel in a post from 2024:

*“Israeli flag for example is a nazi flag for zionists.* [*sic*!]*“.*

Such Nazi analogies can also be found in the form of avatars. For example, a user, who, according to his profile, is from Germany, used a modified Israeli flag as his avatar: A swastika is depicted in the center of the Star of David. The blue stripes of the flag are decorated with the words “*ZIONAZI*” and “*TERRORIST STATE*.”

Overall, antisemitism on Mod DB not only takes various forms, but also crosses ideological boundaries. It is present across different political and ideological spectrums.

### Mixed-ideologies

Our dataset also includes other ambiguous posts and user profiles, which could not be classified as a specific ideology. Mixed and unclear ideological beliefs, i.e., individuals who either pick and choose elements of different (sometimes opposing) ideologies to build their own extremist worldview or individuals who switch between different ideologies, are a growing concern in extremism studies (Gartenstein-Ross et al., 2023; Brace et al., 2024; Meleagrou-Hitchens and Ayad, 2023). We found evidence of such “salad bar” ideologies (The Soufan Center, 2021) on Mod DB.

The most prominent mix of ideologies we found was an overlap of right-wing extremism and Islamism. For instance, we were able to locate several profiles sharing right-wing extremist and NS glorifying imagery while, at the same time, commenting favorably on Islamist mods and using Islamist slogans. One profile, for example, was decorated with imperial war flags and Nazi slogans and simultaneously a member of the group *daesh intervention* and posting slogans such as “*Long live Hezbollah!*.” Another user also posted favorably about both right-wing extremism and Islamism, but seemed highly volatile in his allegiances. They shared classic right-wing extremist tropes such as images of Hitler, statements denying the holocaust, and anti-Israel sentiments. At another point in time, however, they posted a mocking picture of Hitler and expressed the belief that Hitler was in cahoots with the Jews. Simultaneously, this user also posted Islamist content, showed an affinity with both the Iranian and the Syrian regimes, and expressed support for Hezbollah and Palestinian resistance groups. However, the user was changing their views about Hamas frequently, sometimes expressing support, while at other times accusing Hamas of being invented by Israel and, therefore, the enemy.

We also found a small subset of users who, according to their own statements, come from Iran, Iraq, Syria, and Lebanon and show affinity to Islamism, Shiism, and Zoroastrianism (an Iranian religion based on the teachings of Zarathustra) as well as National Socialism. They expressed the belief that Hezbollah and Hamas are ‘*righteous fighters*’ in the name of ‘*real Islam’* while also sharing, for instance, right-wing extremist symbols such as swastikas and runes, Wehrmacht- and Hitler-glorifying content as well as antisemitic conspiracy theories such as the belief in a ‘masterplan’ of Jewish people to perpetrate white genocide. In one post Hitler is referred to as the “*father of today’s Europe*” and another commentator said about the swastika: “*What they call it a symbol of hate, we call it a reason to live.”* Many of these users are clearly connected and frequently comment in Persian on each others’ frontpages. Their posts indicate that they believe to be descendants of a superior race and consider themselves to be “*the original people*,” i.e., of Aryan descent, which may explain the overlap between Islamist and National Socialist tendencies the group displays.

This group of users also shared images, drawn by right-wing extremist cartoonist Ben, Zyklon B, Garrison, with the caption *“The World without Nationalism is a Nightmare*.*”* This cartoon clearly illustrates the extent of the “salad bar extremism” of this group, suggesting that Jewish people control just about every group in society this group of users dislikes, including LGBTQIA+ individuals, feminists as well as PoC and Hispanics.

For example one illustration shows an oversized spider-like creature with a Star of David on its head and peppered with antisemitic stereotypes. A group of people representing stereotypical notions of the ‘woke’ left are placed in front of the creature, showing, for instance, racist, anti-feminist and other dehumanizing tropes. Specifically, there are armed BPoC in attack poses, feminists who are portrayed as anti-Christians and homosexual men who are accused of pedophilia.

## Discussion

### Content

We conducted an exploratory analysis of 500 hateful posts on Mod DB to understand more about identity-based hate and extremism on the platform. As the section above illustrates, we were able to locate posts containing a broad spectrum of hate and extremism, including Islamism, right-wing extremism, antisemitism, and mixed ideology. This suggests that Mod DB - and, by extension, potentially other mod forums which have not been examined yet - may host a variety of ideological posts and are therefore relevant for several strands of extremism research. The posts in our sample were openly accessible and relatively easy to find via a simple keyword search. While we examined a mere 500 out of hundreds of thousands of posts and cannot determine how many users (on average) are exposed to such content on Mod DB, the fact that such content was shared in open parts of the forum, often received considerable amounts of interaction (i.e., comments and up- or downvotes), and had not been deleted or moderated for several years (the oldest post in our sample dates from 2008), may hint at the possibility that many users could be exposed to identity-based hate and extremist content on Mod DB.

Prior research into extremist activities in digital gaming spaces suggests that the most extreme content is often shared in private groups and chats (Schlegel and Amarasingam, 2022). Although we purposefully excluded content from (semi-) closed spaces, we found a considerable amount of overt, explicit identity-based hate, extremist narratives, and even calls for violence in public parts of Mod DB. The fact that not even the ‘diary’ of a suspected ISIS fighter was moderated, is particularly telling. This may suggest that users feel relatively safe to share hateful statements and extremist content openly on the platform without fear of repercussions. Again, this may indicate that even users who are not part of extremist groups on the platform may possibly come across such content even without looking for it.

Content-wise, the posts in our sample did not differ decisively from extremism and identity-based hate found in other digital spaces. We encountered mostly familiar narratives, imagery, codes, and symbols found in Islamist, right-wing extremist and antisemitic discourses on other digital platforms. This indicates that well-known discourses and extremist narratives are reproduced on Mod DB without much adaptation to the specific platform environment. With 71% of all posts in our sample classified as antisemitic, antisemitism emerged as a central element of hateful posts on Mod DB. Research on both online and offline extremist groups has shown that antisemitism is a cross-cutting, bridging narrative (Meiering et al., 2020) and a component of many extremist ideologies, including Islamism, right-wing extremism, and left-wing radicalism (Herf, 2023; Radicalisation Awareness Network, 2022). Antisemitic beliefs can serve as a connecting element between different extremist groups and is one of the core elements that unites, for instance, right-wing extremists, Islamists, and individuals with mixed ideology. This trend was mirrored in our sample of posts on Mod DB, which further underlines that identity-based hate and extremist discourses on the platform largely reflect broader discursive patterns of hate-based communication in other spaces.

Regarding content, there are two main takeaways from our exploratory pilot study: Firstly, Mod DB inadvertently hosts several variants of identity-based hate and extremism, which makes the platform relevant for extremism researchers seeking to understand extremist activities and discursive practices in digital gaming spaces. It also suggests that not including mod forums in research efforts on extremist activities in digital gaming spaces is a crucial gap in current research efforts and deserves more attention in the future. Secondly, hate on Mod DB largely echoes well-known extremist narratives, symbols, and tropes that occur consistently across various digital platforms. In other words, identity-based hate and extremist content on Mod DB does not decisively differ from comparable content in other discursive spaces. Based on our analysis, we anticipate that while the extremist narratives shared on mod forums may not differ decisively from extremist content in other digital spaces, mod forums nevertheless possess distinct affordances that should be examined in more detail in future studies, e.g., how the content of extremist mods influences the (types of) content posted and how the modding of extremist fantasies potentially interacts with broader discussions in these spaces.

### Platform environment

Although the narratives, symbols, and ideological content on Mod DB largely reflect hateful content identified in other digital spaces, there are also peculiarities of the platform that deserve a brief discussion. This includes mods, language, hubs of users posting hate-based content on the platform, as well as reflections on (the effectiveness of) moderation activities.

Mod DB is a platform for exchanging information about modifications of digital games. Consequently, we found that user communication, discussions, and ratings of such mods are central to the platform. Judging by the mods with hateful content and comments we identified, modding seems to be an expression of creative control and an exploration of counterfactual possibilities. Therefore, Selepak (2010) reasoning that right-wing extremist modifications are, at least in part, tools to create different versions of the world and act out the wish for alternative realities, seems to be not only supported by our findings on Mod DB but expanded to include mods with Islamist and anti-Israel content. We also found the comment sections of such mods are often highly polarized with some users vehemently rejecting and others enthusiastically approving of the ideological content and identity-based hate conveyed. However, extremist content and identity-based hate was also located in other parts of Mod DB, including groups and forums. Our preliminary analysis therefore suggests that while mods espousing (elements of) identity-based hate, such hate is not solely confined to the discussion and review threads of such mods. Rather, it is spread across different sections of the platform.

We found few linguistic peculiarities on the platform. The user communication largely resembles communication on other social media platforms and is characterized, for instance, by a low regard for correct spelling and popular abbreviations such as *“LMAO.”* References to and use of gamer language were few and far in between, but some users applied gamer slang such as “*frag*” (the killing of virtual enemies) when commenting on real-life events, e.g., the killing of Jewish people. Generally speaking, communication on Mod DB displays a high degree of harshness, often crossing the line into toxicity, with slurs as a prominent feature. The language used also often has misogynistic undertones. In this regard, Mod DB does not differ decisively from other gaming (−adjacent) platforms as toxicity, harsh language, misogyny, and slurs are frequently found in digital gaming spaces (Wallner et al., 2023; Schlegel and Amarasingam, 2022). Additionally, in our sample, central components of communication are irony and a mocking tone. This may be related to trolling or posting ‘for the lulz’ (see methodology section), but could also suggest the deliberate use of ‘dark humor’ and ‘shitposting’ to insert extremist narratives into mainstream discourses in a ‘fun’ and ‘lighthearted’ manner to normalize such ideas (Fielitz and Ahmed, 2021; Nagle, 2017).

Generally speaking, our preliminary findings suggest that extremist and hateful content on Mod DB is often not explicitly related to gaming. While mods with extremist elements espousing identity-based hate draw a particularly high number of approving comments, we found identity-based hate and extremist narratives, symbols, and imagery across various sections of Mod DB. Similarly, while gamer language could be identified in parts of our sample, it is not dominant. Our findings therefore support the analysis of extremist communication and hateful content on other gaming (−adjacent) platforms. The Institute for Strategic Dialogue, for instance, analyzed extremist content on Twitch, DLive, Discord, and Steam. The authors argue that extremist content in digital gaming spaces is by and large not explicitly interwoven with gaming itself and that extremist activities on such platforms may be motivated by factors that lay beyond gaming (Davey, 2021). Based on our exploratory analysis, we believe the same holds true for a lot of hateful content on Mod DB even if mods displaying extremist narratives tend to draw comments approving identity-based hate.

Furthermore, we found that the majority of hateful content we identified was posted by a limited number of users. A small group of users seems to be particularly active and responsible for a large number of extremist posts in our sample. Some of these users also had hate-based user names such as 卐BlitzKrieg卐 or SS-Panzerkommandant. This suggests two things: Firstly, there is apparently a small cluster or hub of users on Mod DB responsible for posting large amounts of hateful content. These users drive the dissemination of extremist narratives, symbols, and imagery on the platform. Secondly, and just as importantly, this also indicates that the vast majority of users on Mod DB do *not* post such content and are not involved in openly displayed, explicit hate. There seems to be a core group actively posting hate-based narratives, and while we cannot rule out the possibility that many users will be exposed to such content, our sample suggests that most users on Mod DB do not *participate* in such activities.

Lastly, similar to many other digital gaming spaces, we found evidence to suggest relatively poor moderation efforts on Mod DB in comparison to, for example, mainstream social media platforms. We were able to locate what seemed to be community guidelines stating, for instance, “You must not abuse, harass, stalk, threaten, defame or otherwise violate the legal rights (such as rights of privacy and publicity) of others in any form.” It remained unclear whether these guidelines were applied across all of Mod DB by the moderation team. We could not locate any information regarding moderation practices or the moderation team. While we found a few accounts that have apparently been blocked from posting, by and large hateful content and user profiles do not seem to be subject to moderation on Mod DB. Our sample included posts directly attacking other users, including death wishes, as well as openly sharing explicitly extremist content, including well-known extremist symbols and even calls for violence as described above. Many of these posts are several years old, which suggests limited moderation of such content on Mod DB. In a small subset of our sample, we also found evidence to suggest that a few users actively seek to circumvent moderation efforts. This was particularly evident for user profiles posting in both English and Arabic as several users posted largely in English but switched to Arabic when making particularly drastic statements. Nevertheless, even moderation efforts for English language posts seem to generally be poor and ineffective.

Overall, this exploratory pilot study showed that several types of identity-based hate and extremist content are shared on Mod DB, highlighting the relevance of the platform for various strands of extremism research, including research on Islamism, all categories of right-wing extremism, antisemitism, and mixed ideology. It also indicates that the hateful and extremist content shared on Mod DB often does not differ significantly from similar content on other digital platforms and that well-known discourses are reproduced on Mod DB, suggesting that such discourses are even more widespread across various digital gaming spaces than previously known. Such discourses are not necessarily (explicitly) related to gaming, although many relevant posts in our sample were found under mods with hateful elements. While the present study relied on a relatively small sample of posts from a single platform, the results presented above suggest that further research into extremist activities and hateful content on mod forums can support our understanding of the spread and discussion of such content in digital gaming spaces.

## Conclusion

While recent years have seen a stark increase in research on extremist activities in digital gaming spaces, hateful and extremist content shared on mod forums has not been examined so far. Considering the popularity of mods and mod forums in gaming communities, this is a crucial gap in our current understanding of extremist activities in digital gaming spaces. This exploratory pilot study of 500 posts on Mod DB aimed to provide a first glimpse into extremism-related communication in mod forums. It revealed that the platform hosts a range of hateful and extremist content, including Islamist, right-wing extremist and white supremacist, antisemtic and mixed-ideology statements and imagery. Most of this content mirrored hateful posts in other digital spaces and we found few explicit links to gaming in both content and language of the posts in our sample. Our results showed that a considerable amount of identity-based hate and extremist content was posted by a relatively small number of users, which indicates the presence of a small but active hub of users responsible for sharing such content on Mod DB. While we cannot judge whether users posting such content are radicalized, our findings clearly demonstrate that extremist content and identity-based hate are present on Mod DB. This content was publicly accessible, could be easily located via a keyword search, and has been on the platform for years, which suggests a lack of (effective) moderation efforts. Overall, our analysis suggests that mod forums may inadvertently host identity-based hate and extremist content, making them highly relevant for extremism research generally and, in particular, for our understanding of the ecosystem of gaming-related digital platforms containing extremist content.

Our findings open several avenues for future research efforts. Firstly, mod forums deserve more attention from extremism researchers. The analysis presented above indicates that not merely the hateful mods as such but discussions and user communities surrounding modding are of interest and can further our understanding of extremist content and activities in digital gaming spaces. Mod forums should be included in future analyses to ensure a holistic understanding of the ecosystem of gaming-related platforms used to share identity-based hate and extremist content. We would particularly welcome studies that further explore the linkages between hateful comments and individual mods with hateful and extremist content, e.g., by analyzing both the mods as such and the discussion such mods elicit together. Secondly, while we were limited to publicly accessible information, future research should also examine discussions in (semi-) private chats and groups on mod forums as, generally, users share the most extreme content in closed digital (gaming) spaces (Schlegel and Amarasingam, 2022). Thirdly, we purposefully cast a wide net in this exploratory study to examine a broad range of phenomena, but future studies could and should investigate each of these types of content as well as related content such as conspiracy narratives and inceldom in more detail to bring nuance to our understanding of hateful discussions in digital mod spaces. Lastly, it would be beneficial for future research efforts to examine the role mod forums play in user journeys into digital extremism and potential implications for our understanding of radicalization processes in digital gaming spaces.

## Data Availability

The datasets presented in this article are not readily available because restrictions apply to the dataset for this study: the dataset presented in this article is not readily available because it cannot be shared under the RadiGaMe data protection agreement (subject to German data protection laws). Requests to access the datasets should be directed to Linda Schlegel, schlegel@prif.org.

## Footnotes

^1^ https://store.steampowered.com/

^2^ https://www.nexusmods.com

^3^ https://www.moddb.com

^4^ https://www.indiedb.com

^5^ https://www.curseforge.com/

^6^ https://gamebanana.com

^7^ https://www.discord.com

^8^ https://www.twitch.tv

^9^ https://www.roblox.com
